# Identifying Treatments for Taste and Smell Disorders: Gaps and Opportunities

**DOI:** 10.1093/chemse/bjaa038

**Published:** 2020-06-18

**Authors:** Joel D Mainland, Linda A Barlow, Steven D Munger, Sarah E Millar, M Natalia Vergara, Peihua Jiang, James E Schwob, Bradley J Goldstein, Shannon E Boye, Jeffrey R Martens, Donald A Leopold, Linda M Bartoshuk, Richard L Doty, Thomas Hummel, Jayant M Pinto, Casey Trimmer, Christine Kelly, Edmund A Pribitkin, Danielle R Reed

**Affiliations:** 1 Monell Chemical Senses Center, Philadelphia, PA, USA; 2 Department of Cell & Developmental Biology, Rocky Mountain Taste and Smell Center, University of Colorado, Anschutz Medical Campus, Aurora, CO, USA; 3 Center for Smell and Taste, Department of Pharmacology and Therapeutics, 1200 Newell Drive, University of Florida, Gainesville, FL, USA; 4 Black Family Stem Cell Institute, Icahn School of Medicine at Mount Sinai, New York, NY, USA; 5 Department of Ophthalmology, Sue Anschutz-Rodgers Eye Center, University of Colorado School of Medicine, Aurora, CO, USA; 6 Department of Developmental, Molecular and Chemical Biology, Tufts University School of Medicine, Boston, MA, USA; 7 Department of Head and Neck Surgery and Communication Sciences, Duke University School of Medicine, 40 Duke Medicine Cir Clinic 1F, Durham, NC, USA; 8 Department of Ophthalmology, University of Florida College of Medicine, Gainesville, FL, USA; 9 Division of Otolaryngology Head and Neck Surgery, University of Vermont Medical Center, Burlington, VT, USA; 10 Department of Food Science and Human Nutrition, Center for Smell and Taste, University of Florida, Gainesville, FL, USA; 11 Smell and Taste Center and Department of Otorhinolaryngology: Head and Neck Surgery, Perelman School of Medicine, 3400 Spruce Street, University of Pennsylvania, Philadelphia, PA, USA; 12 Department of Otorhinolaryngology, Smell and Taste Clinic, Technische Universität Dresden, Fetscherstrasse, Dresden, Germany; 13 Section of Otolaryngology—Head and Neck Surgery, Department of Surgery, The University of Chicago, MC, Chicago, IL, USA; 14 Firmenich Inc., Plainsboro, NJ, USA; 15 AbScent, Andover, Hampshire, UK; 16 Department of Otolaryngology—Head and Neck Surgery, Thomas Jefferson University, Philadelphia, PA, USA

**Keywords:** cell culture, olfaction, sniff

## Abstract

The chemical senses of taste and smell play a vital role in conveying information about ourselves and our environment. Tastes and smells can warn against danger and also contribute to the daily enjoyment of food, friends and family, and our surroundings. Over 12% of the US population is estimated to experience taste and smell (chemosensory) dysfunction. Yet, despite this high prevalence, long-term, effective treatments for these disorders have been largely elusive. Clinical successes in other sensory systems, including hearing and vision, have led to new hope for developments in the treatment of chemosensory disorders. To accelerate cures, we convened the “Identifying Treatments for Taste and Smell Disorders” conference, bringing together basic and translational sensory scientists, health care professionals, and patients to identify gaps in our current understanding of chemosensory dysfunction and next steps in a broad-based research strategy. Their suggestions for high-yield next steps were focused in 3 areas: increasing awareness and research capacity (e.g., patient advocacy), developing and enhancing clinical measures of taste and smell, and supporting new avenues of research into cellular and therapeutic approaches (e.g., developing human chemosensory cell lines, stem cells, and gene therapy approaches). These long-term strategies led to specific suggestions for immediate research priorities that focus on expanding our understanding of specific responses of chemosensory cells and developing valuable assays to identify and document cell development, regeneration, and function. Addressing these high-priority areas should accelerate the development of novel and effective treatments for taste and smell disorders.

## Introduction

Over 12% of the US population is estimated to experience taste or smell (chemosensory) dysfunction (Hoffman et al. 2016). The chemical senses of taste and smell play a vital role in conveying information about ourselves and our environment. They help identify dangers (e.g., the taste of rancid food and the smell of smoke) and contribute to the daily enjoyment of our food, our friends and family, and our surroundings. Although the loss of taste or smell is not a fatal condition, it affects numerous aspects of human health and has a significant effect on quality of life (Croy et al. 2014; Philpott and Boak 2014; Rawal et al. 2014; Hoffman et al. 2016; Erskine and Philpott 2020).

Chemosensory disorders vary both in their cause and in their consequences for smell or taste functions. Smell disorders are typically divided into 4 categories based on their impact on odor perception. Anosmia is the absence of smell perception, whereas hyposmia indicates a measurably reduced ability to perceive smells. Parosmia is a distortion of smell in which the perceived quality of the smell differs from what it “should” be (e.g., a rose smells like burning rubber). Patients with phantosmia perceive smells even when the odor itself is not present. There are analogous taste disorders, including ageusia (lack of taste function), hypogeusia (reduced taste perception), dysgeusia/parageusia (distorted taste), and phantogeusia (taste phantoms).

Many factors may damage the olfactory or taste systems and reduce function. These factors include trauma, surgical damage, age, bacterial and viral illness, chronic rhinosinusitis, severe allergies, cancer treatments, neurological diseases and disorders, inborn genetic disorders, and some medications (Hummel et al. 2011, 2017). In many cases, the cause of the smell or taste impairment is unknown (idiopathic). Smell disorders resulting from conductive issues, such as nasal polyps, are often treatable with surgical and medical interventions, for example, nasal steroids (Yan et al. 2019). However, biologic therapies for diseases such as nasal polyps are on the horizon (Pinto et al. 2019). Smell training, which involves repeated exposure to odors over weeks or months, is also helpful in some cases (Sorokowska et al. 2017). However, effective treatment interventions are often lacking (Doty 2019). Thus, the search for treatments is important to public health. With this need in mind and with the support of the National Institute on Deafness and Other Communication Disorders (NIDCD; R13 DC017387), 3 major US research centers focused on the chemical senses (the Monell Center, the Rocky Mountain Taste and Smell Center, and the University of Florida Center for Smell and Taste) organized a workshop in November of 2018 to bring together basic and translational sensory scientists, health care professionals, and individuals who have experienced a taste or smell disorder to discuss research opportunities to advance/translate targeted molecular approaches into clinical applications and treatments.

Although our understanding of taste and smell disorders is advancing, participants at the conference identified gaps in scientific knowledge and lack of research capacity, as well as new and innovative research approaches that could facilitate the march toward potential therapies. In addition, participants recognized that the studies of treatments for smell and taste dysfunction comprised a small percentage of ongoing research and would benefit from increased numbers of investigators with training in regenerative medicine entering this scientific area.

In what follows, we summarize the presentations and discussions at the “Identifying Treatments for Taste and Smell Disorders” conference and outline gaps in our current understanding of chemosensory disorders identified by the conference participants. We also discuss potential broad-based research strategies aimed at increasing awareness and research capacity, as well as specific suggestions for immediate research priorities to increase the pace of research, especially in areas likely to lead to therapies.

## Chemosensory disorders

### The “Identifying Treatments for Taste and Smell Disorders” conference

Ninety-four scientists, clinicians, and patients from around the world convened at the University City Science Center in Philadelphia, PA, on November 14 and 15, 2018. The conference was divided into 4 sessions that involved lectures and discussions (Table 1). Organizers met at the conclusion of the meeting to discuss the major themes and potential action items identified over the 2 days.

**Table 1.** Presentations at the “Identifying Treatments for Taste and Smell Disorders” Conference, November 14–15, 2018

Speaker	Affiliation	Talk title
Session I: Stem cell therapies (Chair: Danielle Reed, Monell Center)		
Sarah Millar	University of Pennsylvania	Wnt signaling in taste papilla development, stem cells, and regeneration
Alan Cheng	Stanford University	Regeneration of sensory hair cells in the inner ear balance organs
Natalia Vergara	University of Colorado Denver	3D retinal organoids: new frontiers for regenerative therapies in the eye
Linda Barlow	University of Colorado Denver	Taste cell renewal and cancer therapies
Peihua Jiang James Schwob	Monell Center Tufts University	Stem cells in the taste bud A potential strategy for fixing a broken nose
Bradley Goldstein	Duke University	Olfactory stem cells in the clinic
Session II: Gene therapies (Chair: Linda Barlow, University of Colorado, Denver)		
Jeffrey Holt Shannon Boye	Harvard University University of Florida	Gene therapy in cochlea Gene therapies for *GUCY2D*-associated retinal disease
Jeffrey Martens	University of Florida	Gene therapeutic rescue of congenital olfactory dysfunction
Session III: Clinical Research (Chair: Joel Mainland, Monell Center)		
Donald Leopold Linda Bartoshuk	University of Vermont University of Florida	Smell disorders Taste damage: causes, assessment, treatment
Richard Doty	University of Pennsylvania	Clinical assessment of chemosensory disorders in the U.S.
Thomas Hummel	Dresden University	Clinical assessment of chemosensory disorders in Germany
Jay Pinto	University of Chicago	Epidemiological studies of chemosensory disorders
Session IV: Patient Engagement (Chair: Steven Munger, Unversity of Florida)		
Casey Trimmer	Firmenich	Summary of the scientific sessions for patients
Chris Kelly	AbScent	Patients together on the web: internet resources for chemosensory loss
Edmund Pribitkin	Thomas Jefferson University	Navigating the U.S. health system

#### Session I: Stem cell therapies

The first session focused on stem cells in sensory organs for smell, taste, vision, and balance; the roles of stem cells in normal tissue development and regeneration; and the potential uses of stem cells in modeling complex sensory tissues or in disease therapies (e.g., Ren et al. 2014; Vergara et al. 2017; Gaillard et al. 2019; Kurtenbach et al. 2019; Peterson et al. 2019; Sayyid et al., 2019).

#### Session II: Gene therapies

The second session focused on gene therapy strategies in hearing, vision, and smell (e.g., Gao et al. 2018; Green et al. 2018; McCullough et al. 2019). Gene therapies have shown early success in treating some sensory disorders resulting from single-gene mutations, such as a form of retinitis pigmentosa (Beltran et al. 2012).

#### Session III: Clinical research

On the second day of the conference, patients joined the audience to hear about the latest in clinical research focused on smell and taste disorders (e.g., Snyder and Bartoshuk 2016; Hummel et al. 2017; Bainbridge et al. 2018; Doty 2019; Liu et al. 2019).

#### Session IV: Patient engagement

The final session centered on individuals affected by smell or taste disorders. The session began with a patient-focused summary of the science presented earlier in the conference and a discussion of the challenges that come with navigating the US health system. Importantly, patients and patient advocates shared stories about their own experiences with chemosensory disorders and about ways to engage members of this patient community.

## Our current understanding of chemosensory disorders

The field has made recent progress toward developing methods for quantifying chemosensory dysfunction and establishing definitions of impairment and improvement (Hummel et al. 2017). Yet, despite this progress, many basic questions about taste and smell disorders have yet to be answered. For example, we do not know how to consistently regrow human olfactory neurons or taste receptor cells after injury (e.g., head trauma from sports), illness (e.g., upper respiratory tract infections), or how to reconnect these cells to brain areas that give rise to perception. We do not know how to prevent smell loss after colds, flu or COVID-19, or even why some viral or bacterial infections lead to smell loss and others do not. We do not understand why some people are born without a sense of smell or taste, except in a few rare instances where genetic mutations have been identified (Smith et al. 1965; Pearson et al. 1970; Wolfe and Henkin 1970; Gadoth et al. 1997; Feldmesser et al. 2007; Weiss et al. 2011; Hanchate et al. 2012; Karstensen et al. 2015; Alkelai et al. 2016; Boesveldt et al. 2017; Uytingco et al. 2019). Even in cases where these congenital genetic causes are known, the cures are not. We have no immortalized cell lines for human taste receptor cells or olfactory sensory neurons, so regeneration is hard to study except in bulk tissue or animal models. This lack of knowledge points to a need for greater efforts to answer these questions.

## Suggestions for high-yield next steps

The next steps identified by conference participants involve marshaling the resources to support a more vigorous research effort—both in basic research and in translational studies—to bring discoveries from bench to bedside. The 7 suggestions (Table 2) gathered at the conference fall under the general categories of 1) supporting new avenues of research into cellular approaches, 2) developing and enhancing clinical measures of taste and smell, and 3) increasing awareness and research capacity. Each of these suggestions is discussed below, followed by the specific research priorities that these suggestions indicate.

**Table 2.** Consensus recommendations identified by conference participants

General category	Seven suggestions for high-yield next steps
Support new avenues of research into cellular approaches	1. Cultivate human olfactory and taste cell lines, as well as organoids
	2. Focus on cross-cutting basic principles of sensory regeneration
Develop and enhance clinical measures of taste and smell	3. Build capacity in the analysis of electronic health records and other large-scale data
	4. Disseminate methods to measure taste and smell dysfunction in a clinical setting
	5. Develop new and cost-effective ways to evaluate dysfunction in chemosensory systems
Increase awareness and research capacity	6. Encourage the formation of patient advocacy groups
	7. Train scientists in both stem cell and chemosensory biology

### Support new avenues of research into cellular approaches

Understanding the development, degeneration, and regeneration of taste and smell tissues at the cellular level is necessary to identify sources of sensory dysfunction and to develop treatments to address them. Increasing our focus on cross-cutting basic principles of sensory regeneration that apply broadly to sensory systems will also expand our capacity to develop treatment strategies.

#### Cultivate human olfactory and taste cell lines, as well as organoids

A major reason taste and smell are difficult to study is because one of the tools used in other sensory systems is missing: immortalized cell lines for olfactory sensory neurons and taste receptor cells, particularly of human origin. Investigators have made inroads in primary culture of taste cells (Ozdener and Rawson 2011, 2013; Ozdener et al. 2012) but have not developed immortalized cell lines that are easily shared among research laboratories or that fully recapitulate native cell phenotypes. Progress has also been made in the primary culture of human olfactory stem cells and their differentiation into olfactory sensory neurons, for example, culture expansion of de-differentiated murine basal cells (Goldstein et al. 2016). These olfactory stem cells are being transplanted into model hosts (e.g., mice and rats) to test their ability to recolonize olfactory tissues (Peterson et al. 2019). A primary cell culture approach can be used to identify signaling pathways, molecular targets, and compounds that force olfactory stem cell activation and neurogenesis and, thereby, exploit the endogenous capacity of the resident stem cells of the olfactory epithelium. However, primary cultures may differ from native cells in form and/or function due to their altered environment and the impact of the manipulations needed to culture them. Primary cultures also have a limited lifespan, so immortalized cell lines would be a useful complement, hastening the pace of discovery. For example, immortalized human olfactory cells could be more easily screened in high-throughput systems to identify compounds that would impact cell growth, augment odor responsiveness, or confer resistance to pathogens.

Likewise, taste regeneration is difficult to study in this manner because there are no available immortalized cells. However, alternative strategies are being developed. For example, taste cell organoids—specially grown aggregations of taste receptor cells derived from adult taste stem cells that mimic many qualities of taste buds— are one alternative to immortalized cell lines (Ren et al. 2014; Barlow 2015; Clevers 2016). Taste receptor cell organoids have cell–cell interactions that are especially important for taste cells and, thus, may replicate disease pathology better than traditional cell cultures (Ren et al. 2017). To date, however, no human taste cell organoids have been reported, and the development of both organoid and immortalized taste cell lines would offer significant advantages for developing new therapeutic strategies.

#### Focus on cross-cutting basic principles of sensory regeneration

Harnessing new advances in regenerative medicine, especially those for other sensory systems, is a key pillar in developing treatment strategies for taste and smell disorders. Therefore, an objective of this conference was to invite scientists working on regeneration and therapy in other sensory modalities to learn from their work. They described gene-editing methods to treat single-gene mutations that lead to deafness or blindness (Gyorgy et al. 2019; McCullough et al. 2019), retinal organoid cultures to learn about development and regeneration (Aasen and Vergara 2020), and viral vectors to restore retinal function and useful vision (Gamlin et al. 2019). Similar viral-based gene therapy approaches are also showing promise in restoring auditory or olfactory dysfunctions related to single gene mutations (McIntyre et al. 2012; Williams et al. 2017; Gao et al. 2018).

Decades of studies in cell signaling and development in taste and smell tissues have been crucial in identifying chemical-sensing pathways and molecules, and research with specific olfactory and gustatory progenitor cells is just beginning to yield results. For instance, the core Wnt/β-catenin signaling pathway in stem cells contributes to many sensory systems, including hearing (Jansson et al. 2019) and taste (Gaillard et al. 2015). For olfaction, intranasal stem cell infusion has been at least partially successful at establishing olfactory receptors in mice with hyposmia (Kurtenbach et al. 2019).

Recent progress in these and other areas suggest that taste and olfactory researchers not only will benefit from work in vision and hearing, but also may lead the way in certain areas of regenerative medicine. Benefits may accrue from focusing on the similarities among sensory systems rather than the differences. If we focus too much on the unique properties among sensory systems, we may miss basic principles that will increase the pace of multiple fields. Thus, the “Identifying Treatments for Taste and Smell Disorders” conference represents a valuable step toward developing strong collaborations in regenerative medicine across the sensory fields.

### Develop and enhance clinical measures of taste and smell

Chemosensory loss precedes cognitive deficits in Alzheimer’s diseases (Doty and Hawkes 2019), tremors in Parkinson’s disease (Doty and Hawkes 2019), and cough or fever in COVID-19 (Haehner et al. 2020). Yet, in all of those cases, we learned that the chemosensory loss was associated with the disease well after we learned about the nonchemosensory associations. This is due to a lack of widespread chemosensory testing. In the United States, newborns are screened for hearing before they leave the hospital and vision is checked at primary care appointments to coincide with visual development. In contrast, there is no widespread testing for olfactory function in the general population. In a 2007 survey in the United Kingdom, 97% of consultant otorhinolaryngologists managed olfactory dysfunction, but only 45% formally tested for chemosensory impairment and only 12% did so routinely (McNeill et al. 2007). Fewer than 25% of individuals with smell dysfunction are aware of their sensory deficit until formally tested (Doty 2017) and children with congenital smell loss are typically unaware of the dysfunction until they are over 10 years old (Temmel et al. 2002). Widespread testing would enhance disease diagnosis, aid patients in identifying and addressing chemosensory dysfunction, and help identify the mechanistic underpinnings of disease (Weiss et al. 2011).

#### Build capacity in the analysis of electronic health records and other large-scale data

Although electronic medical health records contain vast amounts of data, we lack standardized methods to measure taste and smell dysfunction across clinical settings, making such information more difficult to analyze. Without such standardized methods, we are limited in our ability to identify early chemosensory impairments as self-report of taste and smell function is poorly correlated with objective tests (Landis et al. 2003). Even when patients present with such impairments, we lack cost-effective ways to evaluate the structural or physiological changes in olfactory or taste tissues underlying these chemosensory deficits or to assess their response to treatment as their sensory capacity recovers.

Analysis of the storehouse of data found in electronic health records has the potential to find new connections between chemosensory dysfunction and recovery. These data are an underutilized source of information about the natural history of taste and smell dysfunction and could be used to understand what patient characteristics are most often associated with a particular type of chemosensory disorder. These types of analysis do not rely on assumptions or prior knowledge about the diseases and conditions and, thus, can identify connections that are missed by traditional types of chart review studies. For example, a recent study found that olfactory loss is a stronger predictor of 5-year mortality rate than heart failure (Pinto et al. 2014). The long observation periods spanned by electronic medical records—potentially from birth to death—and large sample sizes make these databases particularly useful for research on factors associated with rare outcomes like certain types of chemosensory disorders.

Other very large data sets are being generated that can expand the understanding of the etiology and treatment of chemosensory disorders. Some examples are the UK Biobank (Sudlow et al. 2015), the Million Veteran Program (Gaziano et al. 2016), and All of Us (Precision Medicine Initiative Working Group, 2015), which are large-scale efforts to study hundreds of thousands to millions of people through health records, surveys, blood draws, and other medical tests, as well as genetic data. These data sources have the potential to further inform us about the natural history of taste and smell dysfunction and factors associated with recovery. In the United States, the National Health and Nutrition Examination Survey (NHANES) now incorporates questions related to smell and taste alterations, offering yet another resource for querying population data (Rawal et al. 2015; Hoffman et al. 2016; Gallo et al. 2020). When deciding among the available tests to use in large surveys, consider using a test with high reliability coefficients and, when possible, a test that has previously been used in other large surveys to aid in comparison across studies.

#### Disseminate methods to measure taste and smell dysfunction in a clinical setting

A number of sensitive, practical, and well-validated standardized olfactory and gustatory tests have been developed, some with National Institutes of Health (NIH) funding, and are commercially available. Unfortunately, although they are used in academia and in a number of otorhinolaryngology and neurology clinics, their more widespread use has been limited due to lack of physician knowledge and insurance reimbursement. The best known commercially available olfactory tests are the Sniffin’ Sticks test (Kobal et al. 1996), the self-administered University of Pennsylvania Smell Identification Test, and the Brief Smell Identification Test (Doty et al. 1984, 1996), the Japanese T&T olfactometer (Toyota et al. 1978), the NHANES Pocket Smell Test (Rawal et al. 2015; Liu et al. 2016), and the NIH Tool-Box test (Coldwell et al. 2013; Dalton et al. 2013). Some of these tests can be self-administered, so they are practical in clinical settings and, in some cases, can be sent through the mail. Additional clinical tests are also in various stages of development (Hsieh et al. 2017; Douglas et al. 2018; Doty et al. 2019).

#### Develop new and cost-effective ways to evaluate dysfunction in chemosensory systems

Current research and clinical evaluations of taste and smell are hampered by the lack of diagnostic tools that can distinguish the site and type of dysfunction, whether it be from peripheral tissue damage, nerve degeneration, or disruptions to central processing.

Other than an inspection for signs of inflammation, there are few or no methods to judge the health of the taste or smell receptor cells and supporting tissue. Although biopsies are possible for both tissue types and are routinely done for research purposes (Rawson et al. 1998; Spielman et al. 2010), they are too invasive to be part of a routine medical exam. Similarly, recent models for nasal airflow may help clinicians predict surgery outcomes but are not yet at a point where they can be used in routine clinical work (Li et al. 2018).

An additional difficulty is determining if healthy nerves connect peripheral olfactory or taste tissues to the brain. Electroencephalograms can measure responses at the olfactory bulb (Iravani et al. 2020), fMRI methods can evaluate how the brain responds to tastes and odors (e.g., Bao et al. 2016; Iannilli et al. 2019), and intracranial electroencephalograms can assess olfactory function but would typically only be employed in patients with medically resistant epilepsy (Zelano et al. 2016; Jiang et al. 2017). However, even if medically warranted, most of these techniques are expensive, time consuming, and rarely, if ever, are part of the standard medical evaluation of chemosensory dysfunction.

An emphasis on new diagnostic methods to assess the health of smell and taste tissue is critical because therapies cannot be generated without identifying the source of the problem. For instance, growth factors may be required to stimulate the reconnection of axons running through the cranium in cases where smell impairment arises from damage to the olfactory nerve. This is a different strategy than the one that is needed for someone born without an olfactory bulb.

The fluids relevant to each tissue type (saliva in the case of taste and mucus in the case of smell) are also critical for chemosensory function. However, there has been little progress in analyzing these fluids for biomarkers related to inflammation or cellular dysfunction (e.g., Yoshikawa et al. 2018). Researchers can measure inflammation markers in nasal mucus using specialized tests (Pearlman 1999), but no test is available for routine clinical testing.

Measures of tissue health or damage are also critical for evaluating the progress of therapies. Although the restoration of sensory function is the goal and, thus, the ultimate measure of improvement, the effects of technologies, such as stem cell therapy and gene therapy, will likely be incremental. We need objective measures of tissue and organ restoration to tell investigators, and ultimately clinicians, that they are headed in the right direction even before patients experience a noticeable improvement in their sensory ability.

### Increase awareness and research capacity

Increasing awareness of the devastating effects of taste or smell dysfunction can garner public and financial support, a fundamental requirement for significant research to continue. It also brings greater awareness to researchers in related fields and to newly developing researchers, making collaborations and specializations in these fields more likely, which will increase the pace of discovery.

#### Encourage the formation of patient advocacy groups

One path for commanding more research capacity is the use of patient groups as advocates to contribute to public awareness and elicit public and financial support. Patients can become advocates and provide expert knowledge and insights into the needs of people living with chemosensory disorders. These advocates can be key communicators between researchers, funders, and patients on the needs for and results of research into their conditions. Indeed, advocacy groups, such as the National Federation for the Blind and the Hearing Loss Association for America, have been critical advocates for sensory disorders. Similarly, charities such as AbScent (abscent.org/), Fifth Sense (fifthsense.org.uk), and Reuksmaakstoornis (https://reuksmaakstoornis.nl/) have emerged to begin to fill this gap. Patient support groups served through websites and social media allow advocacy groups to reach large audiences. These groups may take on even more international importance with the emergence of chemosensory loss as a symptom of COVID-19 (Pellegrino et al. 2020). Due to national differences in legal requirements for charities and other nonprofit groups, distinct or affiliate advocacy groups will likely need to emerge in each country.

Patient advocates can serve on scientific advisory boards, participate in scientific workshops, and give interviews and presentations about their experience of smell or taste dysfunction. Patient groups can increase the speed of research and discovery by advocating at local, state, federal, and international levels for additional funding. They can also increase research capacity, for example, by organizing registries of people who have taste and/or smell dysfunction who would like to take part in research that would give clinical insights into their disease.

Being private citizens with taste or smell dysfunction gives these advocates credibility with those who allocate research funds. The very formation of the NIDCD, the US National Institutes of Health’s institute devoted in part to the study of deafness and other communication disorders (including smell and taste), is an example of the power of focused patient advocacy (National Institute on Deafness and Other Communication Disorders 2019). Patient advocates can also advise on how to communicate with other patients about the research being done, combating the idea that no one cares and nothing is happening. One example where patient advocacy groups may serve an important role is to work with clinicians to establish insurance reimbursement for routine and specialized care for the sense of taste and smell, a current practical gap in the clinical care of people with chemosensory problems.

#### Train scientists in both stem cell and chemosensory biology

Human embryonic and induced pluripotent stem cells (Thomson et al. 1998; Takahashi et al. 2007) have the potential to transform not only our understanding of taste and smell disorders but also our approaches to treating these conditions, and the pace of developing new knowledge about stem cell biology has been frenetic. These cells are a path forward to establish models of taste and smell development and provide an opportunity to design platforms 1) for discovery, to find drugs that increase taste receptor cell or olfactory sensory neuron numbers, and 2) for predictive toxicology, identifying chemicals that are especially toxic to taste and smell receptors and cells, for example, the thyroid treatment methimazole (Hallman and Hurst 1953; Genter et al. 1995; Clark et al. 2015). Most important, pluripotent stem cells represent a source of cells for therapies to treat patients with taste and smell disorders.

The translation of the remarkable potential of stem cells into practice is dependent on the ability of scientists to direct the differentiation of immature cells to the desired cell type(s). Therefore, the field needs scientists who not only are trained in stem cell biology but also have expertise in chemosensory biology, so that they can understand the progression of immature cells into fully differentiated taste and olfactory receptor cell types. Thus, training programs that integrate stem cell and chemosensory biology are needed. Few existing laboratories study taste and smell regeneration, but these rare laboratories provide a valuable foundation on which to build training programs. The ultimate aim is to increase the pipeline of scientists and the number of established laboratories that are especially important resources for this future growth. One practical step to attract students and early-career scientists into this field would be to add symposia at stem cell biology conferences on chemosensory biology that would attract the attention of both developing and established researchers. Alternatively, symposia focused on stem cell biology can be included at chemosensory research conferences, such as the annual meeting of the Association for Chemoreception Sciences and the European Chemoreception Research Organization.

There is also a need for more clinician-scientists trained in otolaryngology who can evaluate patients, collect tissue, and provide leadership in translational research using stem cells, gene therapy, or other therapeutic approaches. Substantially increased efforts are essential to increase exposure to chemosensory disorders into predoctoral and graduate medical education and into specialty training in otolaryngology at all levels. Of particular need is exposure to the impact of smell and taste disorders on patients, improved clinical care for those affected by these disorders, and increased translational and clinical research in these areas. Strategies to increase participation by otolaryngologists and other clinician-scientists more broadly in the study and treatment of smell and taste disorders could include direct engagement with relevant medical licensing groups and professional societies that impact the content of medical curricula and training of specialists in otolaryngology.

## Specific suggestions for research priorities

These preceeding 7 suggestions for high-yield next steps, aimed at increasing awareness and research capacity, developing and enhancing clinical measures of taste and smell, and supporting new avenues of research into cellular approaches, lead to specific research priorities. The following suggestions are aimed at expanding our knowledge of the development, degeneration, and regeneration of taste- and smell-related cells, as well as developing valuable assays to identify and document these cellular effects, to clear the way for regenerative approaches to treat taste and smell dysfunction.

### Increase knowledge of specific responses of taste- and smell-related cells

Restoration of smell and taste function through the regeneration of lost or damaged olfactory and taste receptor cells is a key aim of the research strategy to address taste and smell dysfunction. However, although restoration/repair of the olfactory and taste cells is a focal point, it is likely that relevant molecular triggers and signals involve a much broader cellular landscape that includes support cells, progenitor cells, and other key factors. In addition, the brain must correctly decode the signals provided by the receptors themselves. Research leading to treatments for chemosensory dysfunction must be supported by a strong research program to identify all these relevant factors.

#### Systems approaches to comprehensive olfactory and taste cell-specific data sets

Using “omic” approaches (e.g., genomics and metabolomics) can identify the vast array of transcripts, proteins, and pathways involved in normal capacity for taste and smell, as well as markers of disease related to chemosensory regeneration (Moyer et al. 2009; Nickell et al. 2012; Ibarra-Soria et al. 2017; Sukumaran et al. 2017; Brann et al. 2020).

#### Immunological and cytotoxic responses

Many cases of taste and smell impairment result from immunological responses to disease, or the cellular responses to cytotoxic treatments, such as those used in chemotherapy. Identifying these responses in mammalian sensory cells will identify markers and targets for approaches to chemosensory regeneration (Wang et al. 2009; Cohen et al. 2016).

#### Characterization of cell cycle and differentiation

Cell growth and development are central to healthy and diseased chemosensory tissues (Vermeulen et al. 2003). Characterizing these processes as they directly relate to the identification and propagation of stem cell populations will be key to taste and smell regeneration.

### Develop assays to identify and document cell development

In tandem with expanding our knowledge of specific responses of taste- and smell-related cells, we need to develop assays that will identify and document these aspects of cell development and key biomarkers in cell degeneration and regeneration, as well as measurements for sustainability of tissue transplantation. This would not only support research to address taste and smell dysfunction but also may be developed into clinical tools to allow us to identify problems and measure outcomes.

#### High-throughput assays

Processing of large numbers of samples across a wide range of patients and subjects is necessary to discover novel cellular signals (pathways) that affect stem cell behavior in sensory cell regeneration (Discher et al. 2009). Development of high-throughput assays for this purpose will be necessary to accomplish this monumental task.

#### Small-molecule screens

Historically, small-molecule screens have identified useful ligands for modulating stem cell fate and developmental signaling pathways related to tissue degeneration and regeneration (Ding and Schultz 2005).

#### Assays to assess transplantation

To evaluate feasibility and sustainability of transplanted taste and smell progenitor cells, we need to develop assays to monitor these processes. This is necessary not only to develop techniques to address taste and smell dysfunction but also to evaluate patients for potential transplantation and to follow the outcomes of the treatment as it progresses.

## Summary

The conference on “Identifying Treatments for Taste and Smell Disorders” brought together basic and translational sensory scientists, health care professionals, patients, and patient advocates who indicated the gaps in our current understanding of chemosensory disorders and suggest next steps to hasten our pace of discovery of treatments for these often debilitating conditions. They identified 7 high-yield next steps, aimed at increasing awareness and research capacity, developing and enhancing clinical measures of taste and smell, and supporting new avenues of research into cellular approaches. These broad-based strategies led to 6 specific suggestions for immediate research priorities that will 1) expand understanding of specific responses of taste- and smell-related cells and 2) develop valuable assays to identify and document cell development. Marshaling the resources to support a more vigorous research effort, both in basic research and in early- and later-stage translational studies, will lead to new and innovative approaches toward identifying patients with taste and smell disorders, the sources of those impairments, and advancing and monitoring potential therapies to mitigate their effects. These high-priority research areas, if addressed, will greatly improve the identification and treatment of taste and smell disorders. Such a commitment to bringing taste and smell research funds and focus to parallel those of sight and hearing is overdue.
